# Models for Predicting Time to Sputum Conversion Among Multi-Drug Resistant Tuberculosis Patients in Lagos, South–West Nigeria

**DOI:** 10.3389/fpubh.2018.00347

**Published:** 2018-11-27

**Authors:** Oluwatosin J. Akinsola, Oyindamola B. Yusuf, Olusoji Mayowa Ige, Patrick E. Okonji

**Affiliations:** ^1^Department of Community Health and Primary Care, University of Lagos, Lagos, Nigeria; ^2^Department of Epidemiology and Medical Statistics, University of Ibadan, Ibadan, Nigeria; ^3^Department of Medicine, University of Ibadan, Ibadan, Nigeria; ^4^Department of Research and Innovation, University of Lagos, Lagos, Nigeria

**Keywords:** mixture cure model, sputum conversion time, MDR-TB, log-normal, log-logistic

## Abstract

**Background:** Multi-drug resistant tuberculosis (MDR-TB) develops due to problems such as irregular drug supply, poor drug quality, inappropriate prescription, and poor adherence to treatment. These factors allow the development and subsequent transmission of resistant strains of the pathogen. However, due to the chronic nature of MDR-TB, cure models allow us to investigate the covariates that are associated with the long-term effects of time-to-sputum conversion among multi-drug resistant (MDR-TB) tuberculosis individuals. Therefore, this study was designed to develop suitable cure models that can predict time to sputum conversion among MDR-TB patients.

**Methods:** A retrospective clinic-based cohort study was conducted on 413 records of patients who were diagnosed of MDR-TB and met inclusion criteria from April 2012 to October 2016 at the Infectious Disease Hospital, Lagos. The main outcome measure (time-to-sputum conversion) was the time from the date of MDR-TB treatment to the date of specimen collection for the first of two consecutive negative smear and culture taken 30 days apart. The predictor variables of interest include: demographic (age, gender and marital status) and clinical (registration group, number of drugs resistant to at treatment initiation, HIV status, diabetes status, and adherence with medication) characteristics. Kaplan-Meier estimates of a detailed survivorship pattern among the patients were examined using Cox regression models. Mixture Cox cure models were fitted to the main outcome variable using Log-normal, Log-logistic and Weibull models as alternatives to the violation of Proportional Hazard (PH) assumption. Akaike Information Criterion (AIC) was used for models comparison based on different distributions, while the effect of predictors of time to sputum conversion was reported as Hazard Ratio (HR) at α_0.05._

**Results:** Age was 36.8 ± 12.7 years, 60.8% were male and 67.6% were married. Majority of the patients (58.4%) converted to sputum negatives. Patients who were resistant to two drugs at treatment initiation had 39% rate of conversion than those resistant to at least three drugs [HR: 1.39; CI: 0.98, 1.98]. The likelihood of sputum conversion time was shorter among non-diabetic patients compared to diabetics [HR: 0.55; CI: 0.24, 0.85]. The overall median time for sputum conversion was 5.5 (IQR: 1.5–11.5). In the cure model, resistance to more drugs at the time of initiation was significantly associated with a longer time to sputum culture conversion for Log normal Cox mixture [2.06 (1.36–3.47)]; Log-logistic Cox mixture cure [2.56(1.85–4.09)]; and Weibull Cox mixture [2.81(1.94–4.19)]. Diabetic patients had a significantly higher sputum conversion rate compared to non-diabetics; Log-normal Cox mixture [2.03(1.17–3.58)]; Log-logistic Cox mixture cure [2.11(1.25–3.82)]; and Weibull Cox mixture [2.02(1.17–3.34)]. However, Log-normal PH model gave the best fit and provided the fitness statistics [(−2LogL: 519.84); (AIC: 1053.68); (BIC: 1078.04)]. The best fitting Log-normal PH model was Y = 1.00X_1_+2.06X_2_+0.98X_3_+2.03X_4_+ε where Y is time to sputum conversion and Xs are age, number of drugs, adherence, and diabetes status.

**Conclusion:** The models confirmed the presence of some factors related with sputum conversion time in Nigeria. The quantum of drugs resistant at treatment initiation and diabetes status would aid the clinicians in predicting the rate of sputum conversion of patients.

## Introduction

Tuberculosis (TB) is second to HIV/AIDS as the greatest killer worldwide due to a single infectious agent. “Nigeria is now the 3rd highest TB country in the world and the first in African region” was among the 22 high burden countries with an annual incidence of 338 per 100,000 and prevalence of 322 per 100,000 individuals (1). According to the National TB Prevalence Survey in 2012, the report also confirmed a worrisome situation with regards to Multi-Drug Resistance Tuberculosis (MDR-TB). Consequently, “Nigeria is now the 13th highest MDR-TB country globally and 2nd highest in the Africa region” (1). In 2014, an estimated 480,000 new cases of MDR-TB occurred and about 190,000 people died of MDR-TB. Since then MDR-TB has emerged as a worldwide problem with an estimated incidence of 425,000 cases occurring annually and the worldwide prevalence estimated to be 2- to 3- times the incidence (1). Yearly, about 8 million people were infected with tuberculosis with an estimated 1.8 million casualties, despite extensive vaccination and drug treatment programmes. In Nigeria, the estimated number of patients with multi-drug resistant tuberculosis (MDR-TB) varies between 2,700 and 4,500 while the prevalence rate of MDR-TB was 2.9% among new patients and 14.5% among previously treated cases in Nigeria (2).

Several studies have consistently reported that different socio-economic problems have influenced outcomes of patients with tuberculosis condition. However, very few of these studies, particularly in Nigeria and in Africa have studied any correlates and prognostic differentials for predicting time to sputum conversion among MDR-TB patients. The knowledge of the correlates and prognostic differentials of sputum conversion time of multi-drug resistance would provide insight into cause and timings of the relapse and factors that influence drug failure. This would help considerably in the management of patients with tuberculosis condition and facilitate the reduction of the degree, as well as, the frequency of the ailment.

Sputum conversion (which is used to monitor program performance) is one the most important interim indicators of pulmonary tuberculosis treatment outcome, measuring efficacy and identifying the constraints. Culture-based monitoring of MDR-TB patients is used to evaluate treatment efficacy and helps to identify those who remain infectious. The internationally agreed-upon definition of culture conversion is two consecutive negative smear/culture from sputum samples collected ≥30 days apart (3). Early conversion is very important to prevent transmission of MDR-TB, reduce hospitalization time, and reduce cost related to infection control measures. There is also some evidence that delayed sputum conversion is associated with amplifications of drug resistance (4). However, due to the chronic nature of MDR-TB, cure models allow us to investigate the covariates that are associated with the long-term effects of time to sputum conversion among MDR-TB individuals (5, 6). Therefore, this study investigated the survival of tuberculosis patients with the aid of suitable models by predicting the time to sputum conversion.

## Methods

### Study area and period

A retrospective cohort analysis was conducted on 413 records of patients who were diagnosed of MDR-TB and met inclusion criteria from April 2012 to October 2016 at the Infectious Disease Hospital, Lagos. Four hundred and twenty-one patients were recruited for the study. The Cochran's sample size formula was used to calculate sample size for the study: n = Z^2^pq/d^2^

Where n is the estimated sample size calculation

Z = standard normal deviate corresponding to 2-sided level of 1% significance level = 2.33

p = prevalence of MDR-TB among cohort of treated patients = 13.1%^2^

q = 1-p = 1–0.131 = 0.869

d = desired level of precision = 4%

n = 2.33^2^ × 0.131 × 0.869/(0.04)^2^ = 386

Therefore, 7% addition of the calculated sample size was added to make up for patients who met the inclusion criteria. A total of 413 patients which represent 99.5% of the estimated sample size were recruited for the study.

### Inclusion criteria

- Patients who resided in Lagos and Ogun axis who were diagnosed of MDR-TB within the period of 2012–2016 and scheduled for routine clinic intervention within Infectious Disease Hospital (IDH), Lagos
- Patients who were ≥18 years with comorbidities such as HIV+ and diabetes

### Data collection method and tool

The main outcome measure (time to sputum conversion) which was used to monitor program performance was thetime from the date of MDR-TB treatment start or date of making diagnosis to the date of specimen collection for the first of two consecutive negative smear/culture taken 30 days apart. Time was computed as the period of months each patients was measured for sputum conversion and *outcome* was the indicator variable (1 when the events of interest are observed and o when censored). The independent variable of interest included age, gender, site of the disease, alcohol intake, smoking status, medical compliance, family history, diabetes status, state of disease (pulmonary/extra pulmonary), registration group [new, relapse, return after default, category four (CAT IV) failure and drug-resistant TB]. In addition, variables such as Human Immuno-Deficiency Virus (HIV) status, patients status: (transferred in, previously exposed with both first line and second line anti-TB drugs and others), type of test (smear/culture), history of TB treatment, treatment outcomes (cured, completed, failed, died, defaulted, transferred out), result (Resistant, Susceptible and Contaminated), Anti-Retroviral Treatment (ART) status (Yes/No), bacillary load, and negative sputum smear and culture at the beginning of treatment, drug-resistant pattern at initiation of treatment, treatment initiation period, number of drugs the initial isolate was resistant to at treatment initiation and time in days to initial sputum culture conversion was also extracted.

### Data management and analysis

R statistical software was used in carrying out the Kaplan-Meier Estimation and Modeling. Statistical Package for Social Sciences (IBM) version 20.0 was used to produce life tables in order to give a detailed survivorship pattern among MDR-TB patients and also to assess the effect of other socio-biological factorsusing Cox regression models. The Cox proportional hazard model was used to determine which of the explanatory variables explains differences in time to sputum conversion of patients with multi-drug resistant tuberculosis. Descriptive statistical techniques were employed to examine the distribution of the patients according to some socio-economic and demographic variables of interest. Mixture Cox cure models were also fitted to the data using Log-normal, Log-logistic, and Weibull models (7, 8). The goodness of fit was assessed using the maximum likelihood technique of −2Loglikelihood statistic. Akaike Information Criterion (AIC) was used for model comparison based on different distributions (9, 10), while the effect of predictors of time to sputum conversion was reported as Hazard Ratios at α_0.05_

## Results

### Socio-demographic characteristics

A total of 413 multi-drug tuberculosis records of patients were reviewed (see Table 1). The mean age of the respondents was 36.8 ± 12.7 years. About a third (32.0%) was between 25 and 34 years. A larger percent of the patients (60.8) were male with a Sex ratio of 1.7 years while majority of them (41.4%) had acquired Secondary education. About two-third (67.6%) of them were married and 68.8% have normal Body Mass Index. Most of the participants (47.7%) were of Yoruba ethnic tribe. More than three-quarters (77.7) of the patients reside with Lagos State.

**Table 1:** Demographic characteristics of multi-drug resistant tuberculosis patients.

**Variables**	**Frequency**	**Percentage**
**AGE GROUP (YEARS)**		
15–24	62	15.0
25–34	132	32.0
35–44	103	24.9
45–59	82	19.9
>60	24	5.8
Unknown	10	2.4
Total	413	100.0
**GENDER**		
Male	251	60.8
Female	148	35.8
Unknown	14	3.4
Total	413	100.0
**LEVEL OF EDUCATION**		
None	78	18.9
Primary	127	30.8
Secondary	171	41.4
Tertiary	29	7.0
Unknown	8	1.9
Total	413	100.0
**MARITAL STATUS**		
Single	84	20.3
Married	279	67.6
Separated	19	4.6
Widow/Widower	24	5.8
Unknown	7	1.7
Total	413	100.0
**TRIBE**		
Yoruba	197	47.7
Igbo	73	17.7
Hausa	28	6.8
Others	98	23.7
Unknown	17	4.1
Total	413	100.0
**LOCATION OF PATIENTS**		
Lagos	321	77.7
Outside Lagos	92	22.3
Total	413	100.0
**BODY MASS INDEX**		
Normal	284	68.8
Overweight	103	24.9
Obese	10	2.4
Unknown	16	3.9
Total	413	100.0

Table 2 shows the clinical characteristics of the MDR-TB patients. A larger percentage (86.9%) of the patients was of pulmonary tuberculosis status. The distribution ratio of the registration category of the patients in respect to new and retreatment cases was 1:3. More than half of them (58.4) converted within the duration of study period while among non-converted, 11.4% extended into extensive drug resistant category. A paltry percentage (13.3%) of the patients were HIV positive and on anti-retroviral treatment (11.1%). Less than a fifth of the patients (15.5%) were cured while 7.3% died, 8.2% are lost to follow-up, 9.2% defaulted, and 5.3% relapsed.

**Table 2:** Clinical characteristics of multi-drug resistant tuberculosis patients.

**Variables**	**Frequency**	**Percentage**
**FORM OF TUBERCULOSIS**		
Pulmonary Tuberculosis	359	86.9
Extra Pulmonary Tuberculosis	54	13.1
Total	413	100.0
**REGISTRATION CATEGORY**		
New	94	22.8
Retreatment	319	77.2
Total	413	100.0
**CONVERSION STATUS**		
Converted	241	58.4
Not Converted	172	41.6
Total	413	100.0
**XDR-TB**		
Yes	47	11.4
No	79	19.1
Unknown	46	69.5
Total	172	100.0
**HIV STATUS**		
Positive	55	13.3
Negative	307	74.3
Unknown	51	12.4
Total	413	100.0
**ANTI-RETROVIRAL TREATMENT**		
Yes	46	11.1
No	308	74.6
Unknown	59	14.3
Total	413	100.0
**COLONY COUNT AT INITIAL CULTURE**		
1+ or 2+	164	39.7
3+ or 4+	172	41.6
Unknown	77	18.7
Total	413	100.0
**BACILLOSCOPY BEFORE TREATMENT**		
Positive	303	73.4
Negative	49	11.9
Unknown	61	14.8
Total	413	100.0
**CLINICAL OUTCOME**		
Treatment completed	204	49.4
Died	30	7.3
Defaulted	38	9.2
Relapses	22	5.3
Loss to follow-up	34	8.2
Cured	64	15.5
Transferred out	21	5.1
Total	413	100.0

### Factors associated with time to sputum conversion among multi-drug resistant tuberculosis patients

Factors associated with time to sputum conversion among MDR-TB patients are as shown in Table 3. The overall median time for sputum conversion was 5.5 (IQR: 1.5–11.5) among those who converted. The median sputum conversion time of patients who resided within Lagos was 3.5 (IQR: 1.5–6.0) months compared to 5.5 (IQR: 3.0–8.5) among those who resided outside Lagos [*p* = 0.037]. In respect of age, the median sputum conversion time for patients who were ≤ 40 years was 4.5 (IQR: 2.0–11.5) compared to 5.25 (IQR: 2.33–9.5) among patients who were older than 40 years [*p* < 0.001]. Also, the median sputum conversion among patients who were resistant to at most two drugs at treatment initiation was 3.75 (IQR: 2.9–6.2) compared to 5.5 (4.6–9.8) among patients who were resistant to at least three drugs [*p* = 0.002]. The median sputum conversion time for patients who adhered to drug medication was 3.5 (IQR: 1.5–6.5) compared to 6.5 (IQR: 2.0–11.33) among patients who did not adhered to drug medication [*p* < 0.001]. Also, the median sputum conversion time for diabetic patients was 6.67 (IQR: 2.5–10.5) compared to 3.33 (IQR: 2.0–6.0) among non-diabetic patients [*p* < 0.001].

**Table 3:** Factors associated with time to sputum conversion among MDR-TB patients.

	**No of patients**	**Converted**	**Not converted**	**Medium duration of conversion (months)**	***P*-value (log-rank test)**
**Variables**		**Percentage**	**Percentage**	
Entire group	413	58.3	41.7	5.5 (1.5–11.5)	NA
Location					0.037^**^
Lagos	321	59.1	40.1	3.5 (1.5–6.0)
Outside Lagos	92	55.4	44.6	5.5 (3.0–8.5)
Age of patients					<0.001^**^
≤40 years	265	64.5	35.5	4.5 (2.0–11.5)
>40 years	138	50.0	50.0	5.25(2.33–9.5)
Unknown	10	10.0	90.0	
Gender					0.198
Male	251	64.1	35.9	5.4 (2.25–8.0)
Female	148	66.9	33.1	6.5 (3.75–10.25)
Unknown	14	42.9	57.1	
Reg. group					0.894
New	94	63.8	36.2	5.5 (3.25–9.0)
Retreatment	319	64.6	35.4	5.0 (2.75–7.75)
No of drugs resistant to at treatment initiation					0.002^**^
1–2	60	76.7	23.3	3.75(2.9–6.2)
≥3	353	55.2	44.8	5.5 (4.6–9.8)
Colony count at initial culture					0.073
1+ or 2+	157	58.6	41.4	4.5 (2.5–7.0)
3+ or 4+	164	70.7	29.3	5.25 (3.5–8.0)
Unknown	92	63.0	37.0	
BACILLOSCOPY BEFORE TREATMENT					0.485
Positive	303	64.0	36.0	5.5 (3.75–8.75)
Negative	49	71.4	28.6	4.75 (3.0–8.25)
Unknown	61	60.7	39.3	
Adherence with medication					<0.001^**^
Yes	196	75.0	25.0	3.5 (1.5–6.5)
No	179	41.3	58.7	6.5 (2.0–11.33)
Unknown	38	57.9	42.1	
Diabetes status					<0.001^**^
Yes	70	24.3	75.7	6.67(2.5–10.5)
No	298	71.8	28.2	3.33(2.0–6.0)
Unknown	45	17.8	82.2	
HIV status					0.228
Positive	55	54.5	45.5	5.75 (3.5–9.5)
Negative	307	66.4	33.6	5.25 (3.0–9.0)
Unknown	51	62.7	37.3	

** *Significant p value*.

### Multivariate analysis of factors influencing time to sputum conversion

#### Cox regression model

*cfit* = *coxph(Conv(time,outcome)* ~ *age* + *location* + *drugresist* + *Diabetes* + *Adherence)*

The factors identified to be significantly associated with time to sputum conversion in bivariate analysis were harvested and subjected to multivariate analysis. The result of the multivariate Cox regression analysis for time-to-sputum conversion is shown in Table 4. The dependent variable in Table 4 is conversion status. Patients who were aged ≤ 40 years or more had 18% increased rate of conversion than those who were aged >40 years (OR = 1.18, *p* = 0.361, 95% CI: 0.83, 1.68). Patients who resided within Lagos had 12% decreased rate of conversion than those who resided outside Lagos (OR = 0.88, *p* = 0.456, 95% CI: 0.62, 1.24). Also, patients who were resistant to one or two drugs at treatment initiation had approximately forty-percent (39%) rate of conversion than those who were resistant to at least three drugs (OR = 1.39, *p* = 0.036^**^, CI: 0.98, 1.98). Non-diabetic patients had 55% rate of conversion than diabetic patients (OR = 0.55, *p* = 0.014^**^, 95% CI: 0.24, 0.85). Patients who adhered with medication had about twenty-percent (19%) rate of conversion than non-adherence (OR = 1.19, *p* = 0.263, 95% CI: 0.88, 1.63).

**Table 4:** Test of association of variables with time to sputum conversion in the Cox model for Multi-Drug Resistant TB patients.

**Variables**	**Crude hazard ratio (95% CI)**	***P*-value**	**Adjusted hazard ratio (95% CI)**	***P*-value**
Age of patients		0.001^**^	1.18 (0.83–1.68)	0.361
≤40 years	2.09 (1.38–3.18)		
>40 years	RC		
Location of patients		0.038^**^	0.88 (0.62–1.24)	0.456
Lagos	0.61 (0.38–0.97)		
Outside Lagos	RC		
No of drugs resistant to at treatment initiation		0.002^**^	1.39 (0.98–1.98)	0.036^**^
1–2	RC		
≥3	0.80 (0.58–1.12)		
Diabetes status		< 0.001^**^	0.45 (0.24–0.85)	0.014^**^
Yes	RC		
No	0.13 (0.07–0.23)		
Adherence with medication		< 0.001^**^	1.19 (0.88–1.63)	0.263
Yes	4.26 (2.74-6.61)		
No	RC		

*RC, Reference category*.

** *Significant p value*.

#### Factors associated with time to sputum conversion among MDR-TB patients using mixture cure model

All the Cox PH mixture cure models showed that all the selected covariates had no effect on the sputum conversion time except outcome status of the converted individuals as shown in Table 5. As indicated in the model, the Odds ratios (OR) and (95% CI) for the number of drugs were [2.06 (1.357–3.472)] for Log normal Cox mixture cure; [2.56 (1.852–4.095)] for Log logistic Cox mixture cure and [2.81 (1.943–4.193)] for Weibull Cox mixture cure while the Odds ratios (OR) and (95% CI) for diabetic status were [2.03 (1.172–3.583)] for Log normal Cox mixture cure; [2.11 (1.252–3.824)] for Log-logistic Cox mixture cure and [2.02 (1.168–3.340)] for Weibull Cox mixture cure, respectively. However, amongst the entire model, the Log normal cure model has the best fitted data as it yielded the lowest goodness of fits criteria as shown Table 5.1.

**Table 5:** Factors associated with time to sputum conversion among MDR-TB patients using mixture cure model.

**Variable**	**Log normal cox mixture cure**	**Log logistic cox mixture cure**	**Weibull cox mixture cure**
	**OR (95% CI)**	**OR (95% CI)**	**OR (95% CI)**
Age	1.02 (0.995–1.018)	1.00 (0.985–1.018)	1.00 (0.985–1.015)
Location of patients	1.05 (0.986–1.078)	1.04 (0.980–1.075)	1.02 (0.992–1.086)
Number of drugs resistant to at treatment initiation	2.06 (1.357–3.472)^**^	2.56 (1.852–4.095)^**^	2.81 (1.943–4.193)^**^
Adherence status	0.92 (0.808–1.121)	0.98 (0.803–1.193)	0.95 (0.777–1.164)
Diabetes status	2.03 (1.172–3.583)^**^	2.11 (1.252–3.824)^**^	2.02 (1.168–3.340)^**^

** *Significant CI values*.

**Table 5.1:** Models' goodness-of-fit statistics (time to sputum conversion).

**Fit statistics**	**Log normal cox mixture cure**	**Log logistic cox mixture cure**	**Weibull cox mixture cure**
−2LogL	516.33	521.44	520.63
AIC	1051.72	1056.57	1064.82
BIC	1076.65	1087.03	1091.40

## Discussion

This study provides important information for understanding the development of suitable models that could predict time-to-sputum conversion among MDR-TB patients. The findings can support activities being implemented to decrease the burden of tuberculosis. An overwhelming majority of the patients were male. This is in agreement with the findings of Hovhannesyan and Breeze (11) that there are fewer females than males in cases of multi-drug resistant TB condition. Nigerian women are extremely sensitive about the stigma associated with TB disease and negative social consequences have been shown to be more of importance to women (12–16). This differential could be attributed to biological and epidemiological characteristics, as well as, socio-economic and cultural barriers in access to health care (17). Moreover, studies have shown that women with pulmonary TB are diagnosed on average 2 weeks later than men due to a delay from the health care provider, and in a study on cough patients it was found that men more often than women were asked for sputum specimen (18, 19).

According to this finding, there is an increasing risk of TB drug adverse events when age increases. Sylvere (19) reported that about two-third of the male population were MDR-TB patients. In previous reports, the occurrence of any major side effects has been associated with age, especially amongst the elderly (13). The frequency of adverse reactions has shown to increase in a progressive and direct form in relationship to age. Overall, vulnerability to adverse reactions are more probable at older ages (20–23). This is usually due to high hepatotoxic level caused by significant reduction in clearance rate of metabolized drug agents by the cytochrome P450 enzyme, changes in the hepatic blood flow distribution, as well as, other factors affecting liver function (24). By and large, favorable outcomes can be achieved in co-infected patients using a community-based treatment model when both MDR-TB and HIV disease are treated concurrently and treatment is initiated promptly.

In this study, patients who resided within Lagos had twelve percent decreased rate of time-to-sputum conversion than those who resided outside Lagos. This variation could be attributed to the proximity to the health care facility for patients who reside within Lagos (25). Also, patients who were resistant to one or two drugs at treatment initiation had approximately forty-percent (39%) rate of sputum conversion than those who were resistant to at least three drugs. This finding was consistent with a previous study of Oladimeji et al. (2) on Intensive-Phase Treatment Outcomes among Hospitalized MDR-TB from a Nationwide Cohort in Nigeria. Besides, non-diabetic patients had 55% rate of sputum conversion than diabetic patients while patients who adhered with medication had about twenty-percent (19%) rate of sputum conversion than non-adherence. These results were true reflection of better monitoring mechanism of patients with prognostic factors of MDR-TB in this facility.

Moreover, the quantum of drugs resistant to treatment initiation and diabetes status emerged as significant risk factors, even after controlling for other variables. The results suggested that patients who had fewer numbers of drugs and diabetic status significantly predicted the sputum conversion time for the converted patients. The reasons for this require further investigation. One explanation might be due to misclassification in proper case definition of the MDR-TB patients since the study was purely based on analytical retrospective study. This is consistent with other MDR-TB programs showing that later cohort's outcomes are likely to be different than earlier cohort outcomes (22).

This research provides an Expectation-maximization (EM) algorithm to fit the mixture cure model to the grouped relative survival data. It can fit both a parametric and a semi-parametric mixture cure model. This algorithm utilizes the standard statistical software (R software) to achieve the M-step and is easier to implement than the Newton-Raphson. The EM algorithm is usually stable than the Newton-Raphson method (26) and the convergence of the EM algorithm is generally fast for the grouped survival data. The estimate of the mixture cure model was fitted by making parametric assumptions vis-à-vis; Log-normal, Log-logistic, and Weibull PH Cox mixture cure. The resulting model showed no significant effects on the treatment initiation period among the patients. However, among the entire model, the Log normal PH cure model has the best fitted data as it yielded the lowest goodness of fits criteria.

From some developed models, various studies have proposed and assessed parametric and semi-parametric mixture cure models such as Default time from tuberculosis treatment in the Southern Republic of Benin Using Mixture Cure Model for Survival Analysis (19). This cohort assessed the cured fraction, the conditional probability of default (CPD) from treatment course and identified the risk factors predicting its timing. With Cox proportional hazards (PH), predictors of default time were HIV/AIDS, TB history, and Age. However, with logistic Cox PH mixture cure model, HIV/AIDS, and Age significantly increased the probability of default, whereas TB history significantly reduced default probability from previous TB infection. This study findings suggests that time to sputum conversion model could enhance the compliance of patients with anti-TB treatment where Age, quantum of drugs, and HIV/AIDS status are identified factors.

## Limitation of study

Study findings should be interpreted with caution especially with the interpretation of the cure fraction estimate. This suggest using the mixture cure models in situations where it is clear that a cured group exists and where there is sufficient follow-up beyond the time when most of the events occur. The estimate of the mixture cure model was fitted by making parametric assumptions vis-à-vis; Log-normal, Log-logistic, and Weibull PH Cox mixture cure. The results of the effect of selected covariates on the treatment initiation among the patients that experienced conversion were assessed. The resulting model showed no significant effects on the treatment initiation period among the patients. However, among the entire model, the Log logistic PH cure model has the best fitted data as it yielded the lowest goodness of fits criteria. Moreover, the estimate of the non-mixture cure model for the survival distribution was fitted by making parametric assumptions vis-à-vis; Log-normal, Log-logistic, and Weibull PH Cox mixture cure. Factors associated with treatment initiation period among non-converted MDR-TB patients were modeled. As indicated in the survival component, the Hazard ratios and 95% CI revealed that the age of patients showed significant association in Log normal PH cure model [1.02 (1.005–1.037)] and Log logistic PH cure model [1.02 (1.006–1.036)]. However, among the entire model, the Weibull PH cure model has the best fitted data as it yielded the lowest goodness of fits criteria.

## Conclusion

In conclusion, despite high rate of drug resistance in the studied population, most patients with multi-drug resistant TB achieved sputum conversion within 13 weeks of starting treatment. Factors negatively associated with culture conversion at 2 months can be easily identified either before diagnosis or early in the course of MDR-TB treatment. This may help in better care of individual patients by identifying them early and treating them vigorously. Mixture cure models allow both the cured proportion and the remaining uncured individuals to be modeled simultaneously with incidence and latency portions, respectively. The great advantage of the mixture model is related to the simple interpretations, especially for medical researchers, where we have the proportion of cured and non-cured individuals given directly in the survival function expression.

## Author contributions

OA conceived the study and was involved in the design, analysis, report writing, and drafting the manuscript. OY and OI were involved in the conception, design, and supervised the work. PO was involved in manuscript review. All authors contributed to and approved the final draft.

### Conflict of interest statement

The authors declare that the research was conducted in the absence of any commercial or financial relationships that could be construed as a potential conflict of interest.
